# Probiotics and Phytoantioxidants as Emerging Neuroprotective Strategies: Bridging the Therapeutic Gap in Open Heart Surgery-Induced Brain Injury

**DOI:** 10.3390/biomedicines14030527

**Published:** 2026-02-26

**Authors:** Yen Chu, Kuo-Hsiung Huang, Chi-Nan Tseng

**Affiliations:** 1 Division of Thoracic and Cardiovascular Surgery, Chang Gung Memorial Hospital Linkou Branch, Taoyuan 33305, Taiwan; 2 Laboratory of Cardiovascular Physiology, Chang Gung Memorial Hospital Linkou Branch, Taoyuan 33305, Taiwan; 3 Department of Research and Development, Chang Gung Memorial Hospital Linkou Branch, No. 5, Fuxing St., Guishan Dist., Taoyuan 33305, Taiwan; 4 Department of Laboratory Medicine, Section of Clinical Serology and Immunology, Chang Gung Memorial Hospital, Linkou Branch, Taoyuan 33305, Taiwan; khs586@cgmh.org.tw; 5 Department of Nursing, College of Nursing, Chang Gung University of Science and Technology, No. 261, Wenhua 1st Rd., Guishan Dist., Taoyuan 333324, Taiwan; 6 Graduate Institute of Traditional Chinese Medicine, The Medical College, Chang Gung University, No. 259, Wenhua 1st Rd., Guishan Dist., Taoyuan 333323, Taiwan

**Keywords:** open heart surgery, brain injury, probiotics, phytoantioxidants, oxidative stress, perioperative neurocognitive disorders, neuroprotection, gut–brain axis

## Abstract

Despite its lifesaving role, open heart surgery (OHS) is frequently complicated by neurological injury resulting from cerebral ischemia and reperfusion (I/R) injury, systemic inflammation, and unavoidable oxidative stress. These pathological cascades lead to neuronal death and cognitive decline, which highlights the urgent need for adjunctive neuroprotective strategies. Probiotics and phytoantioxidants have emerged as promising candidates because of their ability to modulate gut–brain axis signaling, reduce oxidative stress, and support vascular repair. Probiotics provide benefits by stabilizing intestinal microbiota, lowering systemic endotoxemia, and enhancing anti-inflammatory cytokine activity, thereby indirectly protecting neural tissue. Phytoantioxidants such as polyphenols directly neutralize I/R-induced reactive oxygen species (ROS), which promote microvascular recovery and neuronal survival. Experimental studies demonstrate the effects of probiotics and phytoantioxidants in reducing excitotoxic neuronal injury and improving neurovascular outcomes, while preliminary clinical observations suggest potential cognitive benefits in surgical populations. Nevertheless, cohort evidence remains scarce, and standardized clinical trials are required to establish optimal dosing, bioavailability, and long-term efficacy. This review emphasizes the translational potential of probiotics and phytoantioxidants as complementary interventions to mitigate brain injury after OHS, addressing a critical therapeutic gap in perioperative neuroprotection.

## 1. Introduction

### 1.1. The OHS-Induced Neurological Burden

OHS remains a critical intervention for advanced cardiovascular pathology, a legacy spanning back to Gibbon’s pioneering performance in 1953. However, despite iterative refinements in surgical technique and extracorporeal technology, neurological impairment persists as a devastating sequela, carrying significant implications for functional recovery and long-term survival [1]. As the surgical demographic shifts toward an aging population with increasing multi-morbidity, cerebral injury has become a dreaded driver of prolonged hospital stays and elevated mortality [2].

Neurological outcomes following cardiac surgery are traditionally bifurcated into two categories: Type 1 complications include severe focal events such as cerebral death, non-fatal stroke, and transient ischemic attack (TIA) [3], whereas Type 2 complications involve global intellectual deterioration and seizures, with perioperative neurocognitive disorders (PND) being the most prevalent manifestation [4,5]. The incidence of these complications is markedly age-dependent, reflecting diminished cerebral reserve and vascular fragility in the elderly [2,3]. While the incidence of overt stroke is reported at less than 1% in patients under 64 years, it peaks between 7% and 9% in those older than 75 [6]. Systematic reviews have documented acute PND rates approaching 60% within the first postoperative week, particularly among elderly patients [7]. Although partial recovery is observed, persistent deficits remain in 25% to 30% of patients up to one year post-surgery, compromising socioeconomic independence and quality of life [8,9].

### 1.2. Current Therapeutic Gap: The Failure of Macro-Management

Despite the implementation of standard neuroprotective strategies during OHS, the incidence of PND remains persistently high, with recent cohort data showing no evidence of significant global reduction over the past decade [5]. Current clinical guidelines primarily address macroscopic risks, such as maintaining mean arterial pressure, optimizing hematocrit levels, and monitoring cerebral oxygen saturation. While these interventions are essential for reducing overt stroke, they have shown only marginal or inconsistent success in preventing the molecular and inflammatory cascades that underpin PND [10,11].

Furthermore, pharmacological strategies including volatile anesthetic conditioning and systemic anti-inflammatory agents like dexamethasone have failed to demonstrate definitive, sustained neurocognitive benefits in large-scale randomized trials [12,13]. The persistence of PND is a direct consequence of current strategies being “single-target” and cerebro-centric. They fail to counteract the systemic pathology involving I/R injury, systemic inflammatory response syndrome (SIRS), and remote organ signaling [13,14,15]. This leaves a substantial and urgent unmet clinical need for safe, mechanistically diverse perioperative interventions [1].

### 1.3. Rationale for Novel Adjuncts: A Gut–Brain Axis Strategy

The inadequacy of conventional strategies compels a shift toward systemic mediators. Evidence suggests that OHS-induced brain injury is intrinsically mediated by systemic inflammation and oxidative stress, which are critically amplified by cardiopulmonary bypass (CPB)-induced gut dysbiosis and resulting barrier dysfunction [16,17].

This hypothesis justifies exploring probiotics and phytoantioxidants as a multi-target approach to modulate core pathology. Probiotics function by restoring intestinal eubiosis and strengthening the gut barrier, thereby reducing the translocation of inflammatory microbial products. Contemporary research now directly links compromised gut microbiota metabolites, such as short-chain fatty acids (SCFAs), to PND incidence [16,17]. In addition, phytoantioxidants, rich in polyphenols, complement this by acting as potent free radical scavengers and activators of endogenous cellular defense pathways, such as the nuclear factor erythroid 2-related factor 2 (Nrf2) pathway, augmenting the brain’s resilience against the oxidative burst following I/R injury [18]. By simultaneously addressing the systemic inflammatory source and bolstering intrinsic cerebral defenses, a combined probiotic and phytoantioxidant strategy may offer a unique opportunity to bridge the current therapeutic gap and mitigate the neurocognitive burden after OHS. Consequently, this review aims to synthesize the current evidence regarding these interventions, utilizing a structured search approach to identify the most impactful clinical and preclinical findings.

### 1.4. Search Strategy and Selection Criteria

This narrative review is based on a comprehensive literature search of the PubMed and ScienceDirect databases for articles published up to January 2026. The search strategy utilized combinations of the following keywords: “probiotics”, “synbiotics”, “polyphenols”, “cardiopulmonary bypass”, “gut–brain axis”, and “perioperative neurocognitive disorders”. We prioritized peer-reviewed randomized controlled trials (RCTs) and high-quality preclinical and clinical studies specifically addressing the intersection of the gut microbiota and neurological outcomes in the context of cardiac surgery. Only articles published in English were included.

## 2. Pathophysiology of OHS-Induced Brain Injury

The neurological sequelae following OHS result from a highly orchestrated, multifactorial pathological cascade that progressively disrupts cerebral homeostasis. A detailed understanding of the interconnected mechanisms driving OHS-induced brain injury (OHS-IBI) is essential for the development of efficacious therapeutic strategies.

### 2.1. The Cardiac Surgery-Brain Injury Cascade

The necessary introduction of CPB and the subsequent surgical procedure initiate a complex sequence of insults culminating in neuronal damage. The primary hemodynamic insults include both global hypoperfusion during CPB, which jeopardizes the brain’s metabolic oxygen delivery, and the release of microemboli (e.g., gaseous or particulate) originating from the surgical site, aortic cannulation, or the CPB circuit itself [15]. These factors lead to cerebral microinfarcts and localized ischemia, contributing significantly to the immediate neurological deficit.

However, the major sustained threat is the phenomenon of I/R Injury. Restoration of cerebral circulation following periods of hypoperfusion paradoxically initiates a massive burst of ROS [16,17]. This oxidative storm overwhelms the brain’s endogenous antioxidant defenses, leading to lipid peroxidation, protein damage, and, crucially, mitochondrial dysfunction [18]. Damaged mitochondria subsequently release cellular pro-apoptotic factors that drive definitive neuronal death.

### 2.2. Systemic Consequences and Amplification

The local cerebral insult is powerfully amplified by a systemic reaction known as SIRS. Contact between blood elements and the non-endothelial surfaces of the CPB circuit activates the complement system, leukocytes, and coagulation pathways [14]. This activation leads to the massive systemic release of proinflammatory cytokines (e.g., TNF-α, IL-1β, IL-6). These mediators actively cross the compromised blood-brain barrier (BBB), fueling neuroinflammation and microglial activation [12]. This inflammatory state directly exacerbates oxidative stress, creating a vicious cycle of inflammation throughout the central nervous system.

### 2.3. The Pivotal Role of the Gut–Brain Axis and Endothelial Dysfunction

A critical component of OHS-IBI is the link between the gastrointestinal tract and the gut–brain axis [16,19]. CPB-induced splanchnic hypoperfusion damages the intestinal epithelium, resulting in a “leaky gut.” This allows the translocation of bacterial products like lipopolysaccharide (LPS) into the systemic circulation. This endotoxemia drives SIRS and promotes endothelial dysfunction, directly compromising the integrity of the BBB [13]. Once the BBB is breached, systemic neurotoxic factors further accelerate microglial activation and neuroinflammation, leading to the cognitive decline observed in PND [19].

## 3. Probiotics as Neuroprotective Agents

### 3.1. Mechanism: Remodeling the Gut Microbiota

Probiotics, defined as live microorganisms that confer health benefits when consumed in adequate amounts, can enhance beneficial bacterial strains such as Lactobacillus and Bifidobacterium. These microorganisms contribute to a balanced gut ecosystem, which is crucial for optimal health. A key mechanism by which probiotics exert neuroprotective effects is through the remodeling of the gut microbiota. Studies have shown that probiotics positively influence gut composition, leading to a reduction in pathogenic or commensal dysbiosis characterized by elevated levels of endotoxin production, particularly LPS, which contributes to systemic and neuroinflammation [19]. Additionally, probiotics stimulate the production of SCFAs, including butyrate, acetate, and propionate. These metabolites, produced by the fermentation of dietary fibers, modulate inflammation, enhance gut barrier function, and promote neuronal health. The anti-inflammatory properties of SCFAs extend to the central nervous system (CNS), potentially mitigating the risk of cognitive decline following OHS [20].

The pathophysiology of PND during cardiac surgery is recognized as a multifactorial process. While gut–brain axis modulation primarily addresses the systemic inflammatory cascade, it is important to note that these biotherapeutic strategies do not directly mitigate the mechanical embolic hit. This includes microemboli or particulate debris generated during aortic manipulation and CPB. Consequently, probiotics and phytoantioxidants should be viewed as adjunctive therapies intended to provide a neuroprotective buffer against secondary neuroinflammation rather than a replacement for surgical embolic protection techniques.

### 3.2. Modulation of Inflammation and Restoration of BBB Integrity

Restoration of BBB integrity is essential for neuroprotection following OHS. BBB dysfunction during CPB facilitates the entry of circulating toxins, contributing to neuroinflammation [21]. Probiotics, through SCFA metabolites, play a pivotal role in BBB repair. Butyrate-producing strains, such as *Lactobacillus rhamnosus* GG and *Bifidobacterium longum*, upregulate tight junction proteins like occludin and claudin-5, reinforcing the BBB structure [22]. Preclinical models demonstrate that *Lactobacillus plantarum* and *Bifidobacterium breve* reduce BBB permeability and improve neurological outcomes in I/R models [23]. In murine sepsis models, supplementation with these strains preserved BBB integrity, reduced Evans Blue dye extravasation, and attenuated systemic cytokine surges, including TNF-α and IL-6 [24].

### 3.3. Perioperative and CNS Models: Preclinical and Clinical Evidence

Preclinical evidence consistently shows that probiotics enhance recovery in I/R and stroke models. In rat middle cerebral artery occlusion experiments, *Lactobacillus rhamnosus* UBLR-58 and *Bifidobacterium breve* UBBr-01 decreased infarct size by approximately 30% [25]. Using a rat model of OHS, probiotic pretreatment altered the cecal microbiome, preserved gut-barrier and BBB integrity, reduced neuroinflammatory cytokines including IL-6 and IL-1β, and improved spatial memory performance in the Morris water maze. These results support a mechanistic link between gut microbiota modulation and protection against surgery-associated neuroinflammation and cognitive impairment [26].

Furthermore, recent evidence from a CPB model demonstrates that a probiotic mixture of *Bifidobacterium longum*, *Lactobacillus acidophilus*, and *Enterococcus faecalis* relieves PND by modulating the kynurenine metabolic pathway. This intervention effectively shifts the metabolic balance from neurotoxic metabolites (e.g., kynurenine) to neuroprotective metabolites (e.g., kynurenic acid), thereby reducing hippocampal neuroinflammation and preserving synaptic plasticity [27].

## 4. Phytoantioxidants in Neuroprotection

### 4.1. Key Neuroprotective Phytochemicals and Bioavailability

Major neuroprotective phytochemicals include resveratrol (grapes/berries), quercetin (onions/apples), curcumin (turmeric), and epigallocatechin gallate (EGCG) (green tea) [28]. However, low bioavailability remains a significant challenge due to poor solubility, rapid systemic metabolism, and limited BBB penetration [29].

Contemporary strategies to enhance cerebral exposure include liposomal delivery [30], phospholipid complexes [30], and co-administration with adjuvants like piperine to inhibit metabolic clearance [31]. Furthermore, advanced sustained-release formulations, such as multilayer chitosan microparticles, are being developed to maintain therapeutic concentrations of these polyphenols over longer perioperative windows [32].

### 4.2. Multi-Targeted Mechanisms

These compounds act as direct scavengers of reactive oxygen species (ROS), lowering the oxidative load during the critical I/R phase of cardiac surgery [33]. Beyond direct antioxidant activity, a primary mechanism of neuroprotection involves the modulation of the gut–brain axis. Recent evidence suggests that phytoconstituents act as gut microbiota modulators, restoring microbial diversity and reducing the translocation of pro-inflammatory metabolites that drive neuroinflammation [34]. Crucially, these phytochemicals function as potent activators of the Nrf2 signaling pathway. By triggering the translocation of Nrf2 to the nucleus, they upregulate the expression of endogenous antioxidant enzymes, including heme oxygenase-1 (HO-1) and NAD(P)H quinone oxidoreductase-1 (NQO1) [35]. Furthermore, polyphenols help maintain mitochondrial membrane potential and limit the release of pro-apoptotic factors, preserving neuronal ATP production and providing a therapeutic road map to combat neurodegenerative damage triggered by cerebral hypoperfusion [36].

### 4.3. Translational Evidence in Cardiovascular and CNS Cohorts

Clinical studies highlight the systemic benefits of resveratrol, with improved delivery systems showing promise in both cardiovascular and neurological protection. In patients with type 2 diabetes, supplementation significantly improved glycemic control, HbA1c, blood pressure, and lipid profile [37]. In cohorts with stable coronary artery disease, resveratrol conferred cardioprotective effects by enhancing left ventricular diastolic function, improving endothelial performance, lowering LDL cholesterol, and preventing unfavorable hemorheological changes [38].

Translating these findings to the surgical setting, a 2025 randomized trial in patients undergoing coronary artery bypass grafting demonstrated that short-term perioperative quercetin supplementation effectively attenuated vascular senescence, improved endothelial function, and significantly reduced the incidence of post-operative atrial fibrillation in male patients [39]. Complementary evidence from advanced delivery formats of curcuminoids has shown reductions in acute myocardial infarction following coronary artery bypass grafting (CABG) [40], while green tea catechins such as EGCG improved postprandial antioxidant capacity and lipid profile in coronary artery disease cohorts [41].

However, extending these findings to cerebrovascular cohorts remains complex. A long-term resveratrol intake has demonstrated benefits in patients with carotid artery stenosis or occlusion. In a retrospective study, daily supplementation improved cognitive performance across visuospatial, executive, and memory domains, while also enhancing cerebral blood flow in multiple regions including the frontal lobe and thalamus [42]. Nevertheless, evidence from large prospective cohorts such as the InCHIANTI study found no association between resveratrol metabolite levels and cognitive decline, stroke incidence, or overall mortality, indicating the complexity of translating preclinical neuroprotective effects into consistent clinical outcomes [43].

## 5. Probiotics and Phytoantioxidants: Synbiotic Potential

### 5.1. Mechanistic Crosstalk

The therapeutic synergy between probiotics and phytoantioxidants is predicated upon a bidirectional biochemical dialogue designed to improve the bioavailability hurdles observed in isolated antioxidant therapies. While pure polyphenol administration has yielded disparate clinical results in acute neural injury, often failing to impact standardized scores such as the NIHSS due to poor systemic absorption [43], the integration of probiotics provides a necessary catalytic bridge. Probiotics facilitate the biotransformation of complex polyphenols by catalyzing deglycosylation and converting parent compounds into low molecular weight bioactive metabolites such as phenolic acids and urolithins. Unlike their parent compounds, these microbial derived metabolites exhibit superior pharmacokinetic profiles. This allows them to penetrate neural and vascular compartments more effectively and bolster systemic antioxidant defenses during the I/R cycles inherent to CPB [44,45]. Conversely, phytoantioxidants function as prebiotic like modulators that selectively enrich the intestinal milieu for beneficial taxa including *Lactobacillus*, *Bifidobacterium*, and *Akkermansia*. This enrichment significantly upregulates the biosynthesis of short chain fatty acids. These metabolites reinforce gut epithelial junctional integrity and attenuate the trafficking of systemic inflammatory mediators toward the central nervous system [45,46].

### 5.2. Integrated Axis Modulation

A synbiotic approach creates a protective loop across the gut–brain–heart axis. On the gut side, probiotics and supportive polyphenols reduce LPS translocation, downregulate kynurenine pathway activation, and restore barrier integrity [45,46]. On the vascular side, phytoantioxidants improve endothelial nitric oxide bioavailability and reduce oxidative stress, which is pivotal during the I/R cycles associated with CPB [44]. Centrally, the increased availability of polyphenol metabolites and the upregulation of endogenous antioxidant pathways (e.g., Nrf2) counteract neuroinflammation and mitochondrial dysfunction.

### 5.3. Translational Evidence in Surgical Cohorts

Clinical evidence supporting probiotic- and polyphenol-based strategies in cardiac surgery has expanded with specific insights into participant demographics and standardized human dosing regimens:

- OHS and Antioxidant Response

In a randomized controlled trial of patients undergoing OHS (*n* = 37), a high-concentration multispecies probiotic was utilized. The intervention consisted of stable lyophilized strains including *Lactobacillus acidophilus*, *Lactobacillus bulgaricus*, *Lactobacillus plantarum*, *Lactobacillus casei*, *Streptococcus thermophilus*, *Bifidobacterium longum*, *Bifidobacterium breve*, and *Bifidobacterium infantis*. Administered at a concentration of 4.5 × 10^11^ CFU once daily for 14 days postoperatively, this regimen significantly improved total antioxidant capacity and neutralized oxidative damage following CPB [47].

- Elderly Cohorts and Neuroinflammation

In a 2025 double blinded trial involving elderly patients (≥65 yr) undergoing major orthopedic surgery (*n* = 190), a standardized probiotic preparation of *Bifidobacterium longum*, *Lactobacillus acidophilus*, and *Enterococcus faecalis* was administered. The dosage was 210 mg × 4 per dose orally, 2 times per day, starting 1 day before surgery to 5 days after surgery. Although this study focused on lower extremity surgery, the significant reduction in PND and proinflammatory cytokines (e.g., IL-6 and IL-1β) provides a vital translational framework for managing the systemic inflammatory response seen in elderly cardiac patients [48,49].

- Synergistic Delivery Systems for Phytoantioxidants and Microbiota Modulation

Although current cohorts have tested phytoantioxidants [42] or probiotics [48] independently, evidence from large prospective studies [43] shows that habitual resveratrol exposure derived from dietary sources such as red wine, grapes, peanuts, and chocolate did not confer neuroprotective or cerebrovascular benefits. This finding emphasizes the need for innovative co-delivery systems that combine probiotics and polyphenols to enhance bioactive metabolite generation and anti inflammatory efficacy, offering a more promising translational pathway than isolated interventions.

- Valvular Heart Disease and Preoperative Priming

In clinical investigations of patients undergoing heart valve replacement, a standardized preoperative probiotic regimen was evaluated for its ability to mitigate gastrointestinal injury. The intervention utilized a triple strain blend consisting of *Lactobacillus rhamnosus* CMCC (B) P0028, *Lactobacillus rhamnosus* CMCC (B) P0029, and *Enterococcus faecium* CMCC (B) P0030 administered as a daily oral dose of 6 × 10^9^ CFU for seven consecutive days prior to CPB procedure (*n* = 52), this strategy significantly reduced markers of acute gastrointestinal injury and stabilized the gut–heart axis against the stressors of CPB-induced inflammation [50].

### 5.4. Combination Therapies: Exploring Synergistic Neuroprotection

Beyond the use of nutraceuticals in isolation, contemporary evidence highlights the potential of integrating these agents with conventional pharmacological therapies. Current review suggests that probiotics and phytoantioxidants may function as potent adjuncts to standard treatments including statins and sodium–glucose cotransporter 2 inhibitor (SGLT2i). This integration is particularly relevant for stabilizing coronary endothelial function and systemic metabolic markers during the perioperative period [44].

Such multifaceted strategies represent a promising frontier in the preservation of perioperative brain health. By targeting distinct and non-overlapping pathways, these combinations provide a dual-layered defense: while conventional medications like SGLT2i and statins optimize systemic hemodynamics and metabolic flux, phytoantioxidants directly neutralize ROS and probiotics modulate the gut–brain axis. This synergy reinforces BBB integrity and attenuates neuroinflammation by engaging specific cytoprotective pathways that remain largely unaddressed by standard pharmacological protocols [51,52].

### 5.5. Limitations of Current Clinical Evidence

Evidence from a randomized, double-blind, placebo-controlled trial involving 190 elderly patients undergoing elective lower-extremity orthopedic surgery demonstrated that a perioperative probiotic regimen significantly decreased the incidence of postoperative cognitive dysfunction (POCD) [48]. This specific intervention consisted of *Bifidobacterium longum*, *Lactobacillus acidophilus*, and *Enterococcus faecalis* at a dose of 210 mg × 4, twice daily and was administered from one day preoperatively to five days postoperatively. These findings provide a robust proof-of-concept for gut–brain axis modulation in a vulnerable surgical demographic; however, their direct translation to the cardiac surgical population remains limited. For instance, current clinical trials exploring probiotic interventions in cardiac surgery are largely pilot scale and feature smaller cohorts, such as those assessing gastrointestinal barrier integrity in a group of 52 patients [50]. While that study reported a shorter ICU stay and reduced systemic inflammation, its findings were restricted by a 7-day postoperative follow up and specific surgical parameters. These criteria included a CPB duration of less than 132 min and lower Cardiac Surgery Score (CASUS) values. Such constraints, combined with heterogeneous definitions of cognitive decline, leave the long-term impact on permanent cognitive recovery unknown. To address these historical inconsistencies, this review adopts the standardized perioperative neurocognitive disorders (PND) nomenclature. Moving beyond outdated definitions is essential for providing a balanced critical appraisal of how these biotherapeutic strategies may influence the recovery trajectories of the elderly surgical population.

## 6. Clinical Translation and Challenges

The transition of perioperative neuroprotective strategies from experimental promise to clinical reality remains a pressing challenge. Despite advances in mechanistic understanding, the field continues to grapple with fragmented evidence and limited long-term validation.

### 6.1. Evidence Gaps and Methodological Heterogeneity

Current clinical studies often prioritize surrogate markers of recovery such as systemic inflammatory cytokines while systematic neuropsychological evaluation remains a secondary focus [53,54]. Assessment strategies are inconsistent; acute delirium screening is typically confined to the intensive care unit, whereas comprehensive testing for PND is rarely standardized [55]. Furthermore, patient heterogeneity encompassing variations in frailty, baseline cognition, and surgical techniques introduces significant confounding factors that are seldom controlled across trials [46].

### 6.2. Safety, Drug Interactions, and Perioperative Vulnerabilities

While probiotics are generally considered safe, rare risks such as bacteremia or sepsis necessitate vigilance in high risk immunocompromised cardiac populations [56]. A more complex challenge lies in the pharmacokinetic interactions of phytotherapeutics. Compounds like resveratrol, curcumin, and quercetin can modulate cytochrome P450 enzymes, potentially altering the metabolism of essential perioperative drugs including anesthetics and analgesics [57,58].

Furthermore, the dual nature of these compounds must be managed:

- Bleeding Risk: Quercetin can interfere with platelet aggregation by inhibiting thromboxane pathways, posing a potential risk for patients on mandatory anticoagulation [59].
- Hepatotoxicity: High dose EGCG and curcumin have been linked to idiosyncratic liver injury in sensitive individuals, requiring pharmaceutical grade purity to ensure safety [60,61].

While probiotics and phytoantioxidants are generally recognized as safe, their clinical integration into the cardiac surgical suite requires a nuanced appraisal of the elderly patient’s physiological state. A primary concern in the OHS population is the potential for drug nutrient interactions. For instance, high dose quercetin (300 mg of quercetin 4′-O-beta-D-glucoside) has demonstrated antiplatelet and antithrombotic activities by inhibiting platelet aggregation and potentially interfering with the arachidonic acid cascade [59]. These effects necessitate rigorous monitoring when such compounds are co administered with dual antiplatelet therapy or warfarin, as they may theoretically potentiate bleeding risks during the early postoperative period.

Furthermore, the perioperative environment of OHS/CPB presents unique vulnerabilities. The systemic inflammatory response and bypass-induced “leaky gut” increase the theoretical risk of probiotic-induced bacteremia or fungemia, particularly in elderly patients with indwelling central venous lines or those who are severely immunocompromised condition. Additionally, the standard use of broad spectrum antibiotics in the ICU may diminish the viability and efficacy of administered live biotherapeutics, necessitating strategic dosing schedules. Therefore, while these strategies offer significant neuroprotective potential, their application must be balanced against the mechanical and pharmacological complexities of cardiac anesthesia and surgery to ensure patient safety.

### 6.3. Regulatory and Standardization Barriers

A central barrier to adoption lies in the regulatory distinction between dietary supplements and live biotherapeutic products. In the United States and Europe, supplements are subject to less stringent oversight, a disparity that compromises clinical reproducibility. Transitioning to medicinal classification requires an investigational new drug application and adherence to pharmaceutical grade manufacturing standards. Without global harmonization of these regulatory frameworks, evidence generated in one jurisdiction may not be directly translatable to another, stalling the development of unified perioperative protocols. Furthermore, the clinical utility of polyphenol antioxidants is constrained by their inherently poor bioabsorption. Recent studies consistently demonstrate that conventional formulations of resveratrol, curcumin, quercetin, and EGCG yield limited systemic availability, whereas advanced delivery systems such as phospholipid complexes [62] nanoparticles [63], microemulsions [64], and phytosomes [65] have the potential to improve bioavailability. This mirrors challenges seen in probiotics, where no specific strains are determined by modern-omics methodologies, poor bioactivity compromises reproducibility and therapeutic consistency.

## 7. Future Perspectives and Conclusions

The integration of probiotics and phytoantioxidants represents a transformative shift in perioperative care for cardiac surgery. By targeting the gut–brain axis and reinforcing systemic resilience, these nutraceutical strategies offer a biologically plausible means to mitigate PND and enhance vascular recovery. As the field moves from experimental validation toward clinical adoption, several future directions are paramount.

### 7.1. Precision Nutraceuticals and Personalized Protocols

Future research must pivot toward personalized perioperative protocols. Advances in metagenomic sequencing allow for the identification of specific microbial signatures that predispose patients to neuroinflammation. Selecting probiotic strains based on an individual’s baseline enterotype could optimize the production of neuroprotective metabolites such as SCFAs. Similarly, the use of advanced delivery systems like phytosomes and nanoemulsions should be tailored to patient specific metabolic profiles to ensure consistent bioavailability of phytoantioxidants during the high-stress environment of CPB.

### 7.2. Multimodal Synergy and Systems Medicine

The next generation of clinical trials should explore the synergy between nutraceuticals and conventional pharmacological agents. Integrating probiotics with established neuroprotective strategies such as SGLT2 inhibitors or specific anesthetic conditioning may provide additive or synergistic protection of the BBB. A systems medicine approach, combining real-time biomarker monitoring with standardized cognitive assessments, will be essential to refine these multimodal interventions and establish their long-term efficacy across diverse surgical cohorts.

### 7.3. Conclusions

In summary, the perioperative period in OHS is characterized by a complex interplay of systemic inflammation, oxidative stress, and gut dysbiosis that collectively threatens neurocognitive integrity. As highlighted in Table 1, phytoantioxidants such as resveratrol, quercetin and curcuminoid demonstrate promising effects on endothelial function, inflammation, and neurocognitive outcomes. Complementing these findings, Table 2 demonstrates the translational potential of probiotic strategies, which modulate inflammatory cascades, enhance antioxidant defenses, and stabilize the gut–heart axis during CBP or hypoperfusion states. Together, these approaches provide a convergent framework for perioperative cerebrovascular protection. While challenges regarding regulatory standardization, optimal dosing, and pharmacological interactions remain, the clinical evidence from recent cohorts suggests a promising signal. By bridging the therapeutic gap in OHS-induced brain injury, these integrated strategies may offer a translational pathway to improve neurological outcomes and ensure a higher quality of life for patients undergoing OHS.

## Figures and Tables

**Table 1:** Clinical evidence of phytoantioxidants in neurovascular and perioperative Protection.

Clinical Population/Design	Phytocompound	Dosage & Duration	Assessment Window & Outcome Definition	Key Outcomes	Ref.
CAD with CABG Surgery (*n* = 97)/ RCT	Quercetin	500 mg/day; 2 days pre-op through hospital discharge	POD 5-7; Endothelial function (FMD) and POAF incidence	↑ Endothelial function; ↓ Incidence of POAF in men	[39]
MI with CABG (*n* = 121)/ RCT	Curcuminoids	4 g/day; 3 days pre-op until POD 5	POD 5; Incidence of acute MI and inflammatory biomarkers	↓ Incidence of MI; ↓ CRP, MDA, and NT-proBNP	[40]
Asymptomatic CASO (*n* = 79)/ Retrospective	Resveratrol	30 mg/day for 221 days	Month 7; Cognitive performance (ADAS-Cog) and CBF	↑ ADAS-Cog scores; ↑ regional cerebral blood flow	[42]

ADAS-Cog: Alzheimer’s Disease Assessment Scale-Cognitive Subscale; CABG: Coronary Artery Bypass Graft; CAD: Coronary Artery Disease; CASO: Carotid Artery Stenosis/Occlusion; CBF: Cerebral Blood Flow; CRP: C-Reactive Protein; FMD: Flow-Mediated Dilation; MDA: Malondialdehyde; MI: Myocardial Infarction; NT-proBNP: N-terminal pro-B-type Natriuretic Peptide; POAF: Postoperative Atrial Fibrillation; POD: Postoperative Day. RCT: Randomized Controlled Trial. *n*: the enrolled subjects”; ↑: increase; ↓: decrease.

**Table 2:** Translational evidence of probiotics and synbiotics in perioperative neuroprotection.

Clinical Population/Design	Probiotic Intervention	Dosage & Duration	Assessment Window & Primary Endpoint	Key Outcomes	Ref.
OHS (*n* = 37)	Multispecies (8 strains)	4.5 × 10^11^ CFU/day; 14 days post-op	POD 14; TAC	↑ TAC; ↓ Oxidative damage post-CPB	[47]
Major Orthopedic Surgery (*n* = 190)/ RCT	Triple strains (*B. longum,* *L. acidophilus*, *E. faecalis*)	210 mg × 4; 2× daily; 1 day pre-op to POD 5	POD 5; PND (dNCR) incidence (Standardized cognitive battery)	↓ PND (dNCR) incidence; ↓ IL-6 and IL-1β	[48]
Heart Valve Replacement (*n* = 52)/ Prospective	Triple strains (*L. rhamnosus p0028*, *L. rhamnosus p0029*, *E. faecium*)	6 × 10^9^ CFU/day; 7 days pre-op	Pre-op to POD 1; Gastrointestinal barrier marker (DAO)	↓ Markers of acute GI injury; stabilized gut–heart axis	[50]

CFU: Colony Forming Units; CPB: Cardiopulmonary Bypass; DAO: Diamine Oxidase; dNCR: Delayed Neurocognitive Recovery; GI: Gastrointestinal; IL: Interleukin; OHS: Open Heart Surgery; PND: Perioperative Neurocognitive Disorders; POD: Postoperative Day; RCT: Randomized Controlled Trial; TAC: Total Antioxidant Capacity. *n*: the enrolled subjects”; ↑: increase; ↓: decrease.

## Data Availability

No new data were created or analyzed in this study.
